# Reply to Cecchi and Palminteri: On the need to model temporal variation in learning rates

**DOI:** 10.1073/pnas.2535857123

**Published:** 2026-02-03

**Authors:** Prakhar Godara

**Affiliations:** ^a^Department of Psychology, New York University, New York, NY 10003

We appreciate Cecchi and Palminteri’s [CP, (1)] interest in our work (2). We begin by clarifying that (2) does not claim that human behavior is best described by models with decreasing learning rates. Indeed, we explicitly document ways in which human behavior deviates from Bayesian predictions (*SI Appendix*, section 4). Our central point is methodological: temporal variation in learning rates can mimic confirmation bias. Therefore, failing to account for temporally varying learning rates before introducing valence-based asymmetries risks overestimating the magnitude of bias.

CP challenge our comparison of Bayesian and biased RL models (Fig. 4*B*), but two methodological choices explain the discrepancy:

1. CP fit a model designed for stationary environments (with learning rate 1/(t+3)) to both stationary and nonstationary environments.
2. CP fit one set of parameters to all episodes of a subject.

Regarding (1), applying a stationary Bayesian update rule to reversal blocks is inappropriate: the model is misspecified by design, so poor fits are expected. Our analysis intentionally restricts itself to stationary environments to isolate the confound between temporal learning-rate variation and confirmation bias. If nonstationary environments are included, Bayesian models with assumed volatility—which also imply time-varying learning rates—provide better fits than asymmetric RL models (3).

**Fig. 1.**
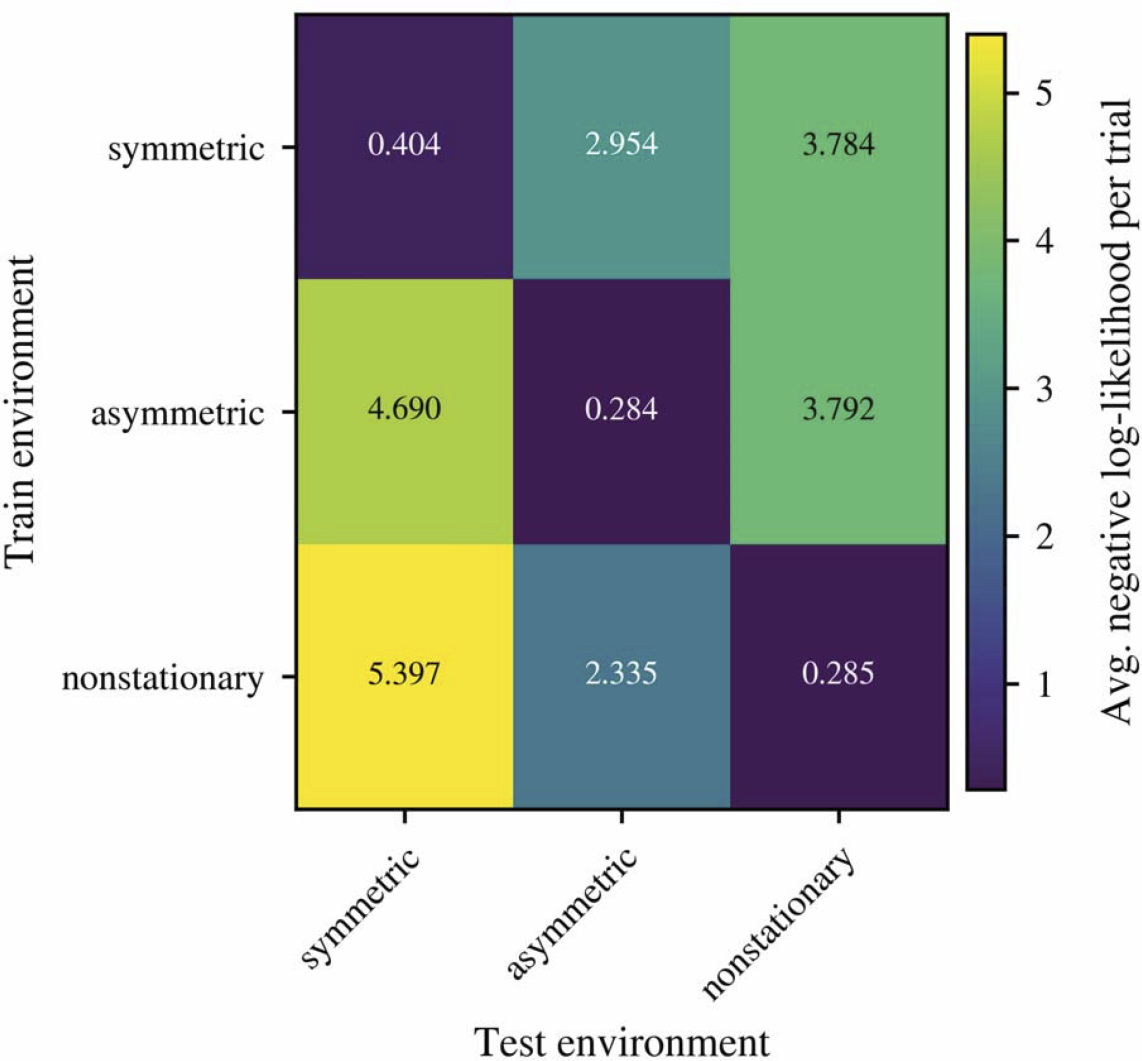
Cross validation scores for the “bias” model across environment types.

Regarding (2), there is no consensus on whether reinforcement-learning parameters should be fit jointly across all episodes of a subject or separately for each episode. Several studies show that RL parameter estimates often exhibit low test–retest reliability (4, 5), and human behavior can vary substantially from block to block due to factors such as attention or fatigue. In our data, parameter reliability across symmetric, asymmetric, and reversal environments is low (Table 1), and cross-validation reveals poor generalization of parameters across environment types (Fig. 1). In light of this variability, we chose to fit parameters separately for each episode, while recognizing that alternative modeling choices are possible.

**Table 1.** Intraclass correlation coefficients ICC(3,1) for each parameter across environment types (5)

	$\alpha_{c}^{+}$	$\alpha_{c}^{-}$	$\alpha_{u}^{+}$	$\alpha_{u}^{-}$	β
ICC(3,1)	0.0471	−0.0077	0.1099	0.1264	−0.0401

ICC values below 0.4 suggest poor reliability.

Finally, CP introduce hybrid models with geometrically decaying, valence-dependent learning rates as evidence that temporal dynamics alone cannot account for behavior. However, these models impose a particular parametric form on learning-rate dynamics. Humans are known to infer environmental volatility, which implies flexible, often nonmonotonic changes in learning rates near change points. Given the wide space of possible temporal dynamics, rejecting one specific form does not negate the broader point: temporal variation must be modeled before attributing behavior to valence-based bias.

All analyses reported here, including parameter reliability and cross-validation tests, are available at: https://github.com/prakhargodara/Bandit-parameters-ICC-and-cross-validation-scores.
